# Expression of Concern: Circular RNA hsa_circ_0072309 inhibits non-small cell lung cancer progression by sponging miR-580-3p

**DOI:** 10.1042/BSR-20194237_EOC

**Published:** 2020-06-24

**Keywords:** hsa_circ_0072309, non-small cell lung cancer, miR-580-3p

The Editorial Office has been made aware of potential issues surrounding the scientific validity of this paper, hence has issued an expression of concern to notify readers whilst the Editorial Office investigates.

